# Editorial: Emerging digital technologies as a game changer in the sport industry

**DOI:** 10.3389/fspor.2025.1605138

**Published:** 2025-04-29

**Authors:** Ekaterina Glebova, Yiran Su, Michel Desbordes, Pierre-Olaf Schut

**Affiliations:** ^1^ CIAMS, Université Paris-Saclay CIAMS, Orsay, France; ^2^ Faculty of Business, Higher Colleges of Technology, Dubai, United Arab Emirates; ^3^ Isenberg School of Management, University of Massachusetts Amherst, Amherst, MA, United States; ^4^ Institut des Sciences du Sport SSP (ISSULssp), Université de Lausanne, Lausanne, Switzerland; ^5^ Analyse Comparée des Pouvoirs (ACP) - EA 3350, Université Gustave Eiffel, Bouguenais, France

**Keywords:** emerging technologies, artificial intelligence, sports, sport industry, technological transformation

**Editorial on the Research Topic**
Emerging digital technologies as a game changer in the sport industry

The sports industry is undergoing a profound transformation, driven by the rapid evolution of digital technologies (1, 2). From fan engagement to athlete performance (3), data analytics (4), and immersive experiences, virtual and augmented reality (5), innovations such as artificial intelligence (Glebova et al.), blockchain (6), and the metaverse are redefining how sport is played, consumed, and commercialized (7).

This special issue explores the disruptive potential of these emerging technologies, offering fresh insights into their applications and implications. The following five articles examine key developments and theoretical perspectives that highlight how digital advancements are reshaping the sports ecosystem.

The first article in this special issue, “*Enhancing Volleyball Training: Empowering Athletes and Coaches through Advanced Sensing and Analysis*” explores how cutting-edge sensing technologies and machine learning are transforming volleyball training. Salim et al. present an innovative system that leverages Inertial Measurement Units (IMUs), a pressure-sensitive display floor, and AI-driven action recognition to provide coaches and athletes with real-time performance insights. Their study introduces two key applications: an automatic video-tagging system that marks key volleyball actions with high accuracy and a “bump-set-spike” trainer that delivers instant feedback to players, optimizing skill development. By integrating wearable sensors and interactive dashboards, this research highlights how technology can create data-driven, adaptive training environments, ultimately enhancing both individual and team performance in volleyball and beyond.

The second article “*Unlocking High-Value Football Fans: Unsupervised Machine Learning for Customer Segmentation and Lifetime Value*” explores how data-driven decision-making can improve a football club's marketing strategy by segmenting fans based on their engagement and spending behavior. Chouaten et al., apply a weighted Recency, Frequency, and Monetary (RFM) model alongside unsupervised learning techniques to analyze 500,591 merchandising transactions from AFC Ajax. The study identifies six fan clusters, ranging from high-value “Golden Fans” to disengaged segments in need of re-engagement strategies. By estimating Customer Lifetime Value (CLV), the authors provide actionable insights for targeted marketing, loyalty programs, and personalized engagement initiatives. Their findings highlight the potential of AI-powered segmentation to strengthen football clubs’ commercial strategies and optimize resource allocation.

The third one, the research report “*Artificial Intelligence Development and Dissemination Impact on the Sports Industry Labor Market*” examines how artificial intelligence (AI) is reshaping the sports industry labor market. Glebova et al. explore the role of AI in creating new job opportunities, transforming existing roles, and introducing challenges related to automation and ethics. Through a literature review and qualitative interviews with industry professionals, the study highlights the increasing demand for AI-skilled sports analysts, marketing experts, and governance specialists while also emphasizing the need for upskilling and ethical oversight. As AI continues to drive innovation, understanding its implications on the labor market is crucial for both industry professionals and policymakers.

The fourth article, a review entitled “*Performance and Healthcare Analysis in Elite Sports Teams Using Artificial Intelligence: A Scoping Review*” provides a comprehensive overview of AI applications in elite sports teams, with a focus on performance enhancement and healthcare management. Munoz-Macho et al. analyze 32 studies to assess how AI is shaping talent identification, game prediction, tactical support, and injury prevention. Their findings reveal that football (soccer) dominates AI research in sports, accounting for 67% of the studies, with various machine learning techniques—Tree-based methods, Neural Networks, and Support Vector Machines—being widely used. This review underscores the growing role of AI in optimizing athlete performance and health management, offering valuable insights for researchers, practitioners, and policymakers.

The fifth and final perspective-format article “*Twin Transformation as a Strategic Approach in Sport Management: The Synergy of Digitalization and Sustainability in Sports*” introduces the Twin Transformation (TT) framework, which highlights the strategic integration of digitalization and sustainability in sport management. Glebova and Madsen explore how TT can drive innovation, enhance fan engagement, and promote environmental responsibility within the industry. By synthesizing theoretical insights and real-world examples, the paper demonstrates how TT can reshape operational practices, stakeholder interactions, and long-term strategic planning in sports. The study also calls for further research to deepen our understanding of the implications of TT and offers valuable perspectives for researchers, practitioners, and decision-makers navigating the evolving sports landscape.

Taken together, these five articles underscore the rapid digital transformation of the sports industry, where AI, data-driven strategies, and sustainable digitalization are shaping training, business strategies, labor markets, and management practices. As sports organizations continue to embrace technological innovation, these studies provide critical insights for researchers, practitioners, and decision-makers navigating this evolving landscape. The insights from this special issue open up several critical avenues for future research, particularly as digital transformation continues to shape the sports industry. Key research directions and emerging themes that could guide future studies:

1. AI-powered training and Performance Optimization
  • Developing personalized AI coaching systems that adapt to the individual needs of athletes.
  • Exploring multi-sport applications of AI-powered training beyond volleyball and football.
  • Investigating the long-term impact of AI-driven feedback on athlete development and injury prevention.
2. Data-Driven Fan Engagement and Consumer Behavior
  • Applying advanced machine learning models to enhance fan experience personalization in digital and physical stadium environments.
  • Exploring blockchain and NFTs for new fan engagement strategies in sports marketing.
  • Investigating the impact of AI-driven segmentation on ticketing, merchandising, and sponsorship effectiveness.
3. AI's Disruptive Influence on the Sports Labor Market
  • Analyzing new skill requirements in sports management as AI automates traditional roles.
  • Assessing the need for retraining and upskilling programs for sports professionals.
  • Investigating the role of ethical AI governance in mitigating bias and ensuring fair employment practices.
4. AI in Healthcare and Injury Prevention
  • Advancing real-time injury prediction models using wearable technology and AI.
  • Exploring the role of AI in the mental health monitoring of athletes and sports professionals.
  • Developing ethical frameworks for the use of biometric data in elite sports performance analysis.
5. The Role of Twin Transformation (Digitalization + Sustainability) in Sports Management
  • Assessing eco-friendly digital innovations in stadium operations, merchandising, and event management.
  • Exploring the circular economy in sports—using technology to reduce waste in apparel and equipment manufacturing.
  • Investigating how sports organizations balance digital growth with sustainability commitments in marketing, logistics, and governance.

Emerging Themes for Future Research

• Human-AI Collaboration (The interplay between human expertise and AI decision-making in coaching, refereeing, and sports management; see (2).
• Extended Reality (XR) in Sports (The impact of AR/VR in training, fan engagement, and immersive experiences; see (5).
• Decentralized Sports Ecosystems (The role of blockchain in athlete contracts, ticketing, and sports governance; see (8).
• AI Ethics and Fairness in Sport (Developing regulatory frameworks to ensure that AI-driven decisions in player scouting, game officiating, and performance evaluation are transparent and unbiased; see (4).

As digital technologies become increasingly embedded in sport, future research must not only push the boundaries of AI-driven innovation but also ensure that these advancements align with ethical, sustainable, and human-centered principles. These research directions will be crucial in shaping the next era of digital transformation in sport.

These five articles do not claim to be exhaustive. Rather, they offer a unique perspective on a reality that is developing at an accelerating pace while at the same time possessing a strong disruptive potential. The sports sector is not the only one impacted, and not in a completely singular way. The digital transformations taking place in other sectors represent potential technology transfers that could very quickly affect the sports sector. Consequently, the reflections shared here are just a glimpse of things to come (9).
